# Psychiatric safety of bimekizumab versus interleukin-23 inhibitors in psoriasis: A real-world TriNetX cohort study

**DOI:** 10.1016/j.jdin.2026.02.006

**Published:** 2026-02-23

**Authors:** Christina Tolete, Adam Friedman, Joaquin Calderon, Leonardo Tjahjono

**Affiliations:** Department of Dermatology, George Washington University, School of Medicine and Health Sciences, Washington, District of Columbia

**Keywords:** bimekizumab, depression, IL-17 inhibitor, IL-23 inhibitor, mental health, psoriasis, suicidal ideation, suicide

*To the Editor:* Bimekizumab, a dual interleukin (IL) 17A/F inhibitor, has demonstrated robust efficacy in moderate-to-severe psoriasis, but long-term safety regarding psychiatric adverse events remains incompletely characterized.^1^ Given prior class concerns from the IL-17 receptor blocker brodalumab, we evaluated whether bimekizumab use is associated with an increased risk of major depressive disorder (MDD) or suicidal ideation (SI) compared with IL-23 inhibitors in real-world practice using the TriNetX US Collaborative Network.^2^

We conducted a retrospective cohort study using the TriNetX platform. We identified adults (aged ≥ 18 years) with psoriasis (International Classification of Diseases, 10th Revision code L40) who initiated bimekizumab, risankizumab, guselkumab, tildrakizumab, or ustekinumab. The index date was defined as the first recorded prescription of the respective biologic, and outcomes were evaluated beginning 30 days after initiation to minimize baseline confounding and evaluated up to 730 days (2 years), reflecting the more recent Food and Drug Administration approval of bimekizumab. The primary outcome was new-onset depression, MDD, or SI defined by International Classification of Diseases, 10th Revision, Clinical Modification codes F32, F33, and R45.851 and use of antidepressants and antipsychotics defined by Anatomical Therapeutic Chemical Classification System code N06A and N05A. Patients with prior depression, MDD, SI, or use of antidepressants and antipsychotics were excluded. Cohorts were propensity score–matched 1:1 for demographics, comorbidities (including obesity, diabetes, hypertension, inflammatory bowel disease, and arthropathic psoriasis), and baseline medication use (glucocorticoids, methotrexate, and cyclosporine). Comparative analyses included risk with statistical significance set at *P* < .05.

After propensity score matching, 3792 patients were included, with 1896 receiving IL-23 inhibitors (risankizumab, guselkumab, tildrakizumab, or ustekinumab) and 1896 receiving bimekizumab. Baseline characteristics were well balanced across cohorts, with a mean age of approximately 51 years, 62% female, and similar distributions by race, ethnicity, and comorbidities including obesity, hypertension, diabetes, inflammatory bowel disease, and arthropathic psoriasis (Table I).

**Table I:** Baseline demographic and clinical characteristics of patients treated with IL-23 inhibitors versus bimekizumab before and after propensity score matching (PSM). Continuous variables are presented as mean (standard deviation [SD]) and categorical variables as number (percentage). Standardized mean differences (SMDs) were used to evaluate covariate balance between treatment groups, with values <0.10 indicating adequate balance. After 1:1 PSM, the IL-23 inhibitor and bimekizumab cohorts were well balanced across demographic characteristics, comorbidities, and prior medication use

Characteristic	Before PSM, *n* (%)			After PSM, *n* (%)		
	IL-23 (*n* = 54,047)	Bimekizumab (1897)	SMD	IL-23 (*n* = 1896)	Bimekizumab (*n* = 1896)	SMD
Age at index, mean (SD)	49.9 (15.7)	50.7 (14.0)	0.050	50.8 (14.1)	50.7 (14.0)	0.006
Sex						
Female	30,647 (56.7)	1176 (62.0)	0.108	1198 (63.2)	1175 (62.0)	0.025
Male	23,375 (43.2)	721 (38.0)	0.107	698 (36.8)	721 (38.0)	0.025
Race						
Asian	1796 (3.3)	101 (5.3)	0.099	81 (4.3)	100 (5.3)	0.047
Black or African American	3111 (5.8)	128 (6.7)	0.041	124 (6.5)	128 (6.8)	0.008
White	43,003 (79.6)	1443 (76.1)	0.084	1497 (79.0)	1443 (76.1)	0.068
Native Hawaiian or Other Pacific Islander	191 (0.4)	12 (0.6)	0.040	10 (0.5)	12 (0.6)	0.014
Comorbidities						
Overweight and obesity	13,975 (25.9)	727 (38.3)	0.269	718 (37.9)	726 (38.3)	0.009
Tobacco use	2351 (4.3)	112 (5.9)	0.071	107 (5.6)	112 (5.9)	0.011
Primary hypertension	18,652 (34.5)	772 (40.7)	0.128	728 (38.4)	772 (40.7)	0.047
Type 2 diabetes	8692 (16.1)	437 (23.0)	0.176	423 (22.3)	436 (23.0)	0.016
Crohn’s disease	3907 (7.2)	16 (0.8)	0.329	10 (0.5)	16 (0.8)	0.038
Ulcerative colitis	2118 (3.9)	12 (0.6)	0.222	10 (0.5)	12 (0.6)	0.014
Arthropathic psoriasis	15,867 (29.4)	1124 (59.3)	0.631	1137 (60.0)	1123 (59.2)	0.015
Medications						
Glucocorticoids	38,552 (71.3)	1601 (84.4)	0.319	1588 (83.8)	1600 (84.4)	0.017
Cyclosporine	2066 (3.8)	136 (7.2)	0.147	115 (6.1)	135 (7.1)	0.043
Methotrexate	10,613 (19.6)	631 (33.3)	0.313	637 (33.6)	630 (33.2)	0.008

*IL-23*, Interleukin-23; *PSM*, propensity score matching; *SD*, standard deviation; *SMD*, standardized mean difference.

Following exclusion of patients with prior depression, SI, or antidepressant or antipsychotic use, 855 IL-23–treated and 836 bimekizumab-treated patients remained for outcome analysis. During follow-up, 12% of IL-23 users and 4.9% of bimekizumab users developed depression, SI, or was taking an antidepressant or antipsychotic. The risk ratio was 2.43 (95% confidence interval: 1.715-3.451), indicating a significantly higher incidence among IL-23 users (Fig 1).

**Fig 1:**
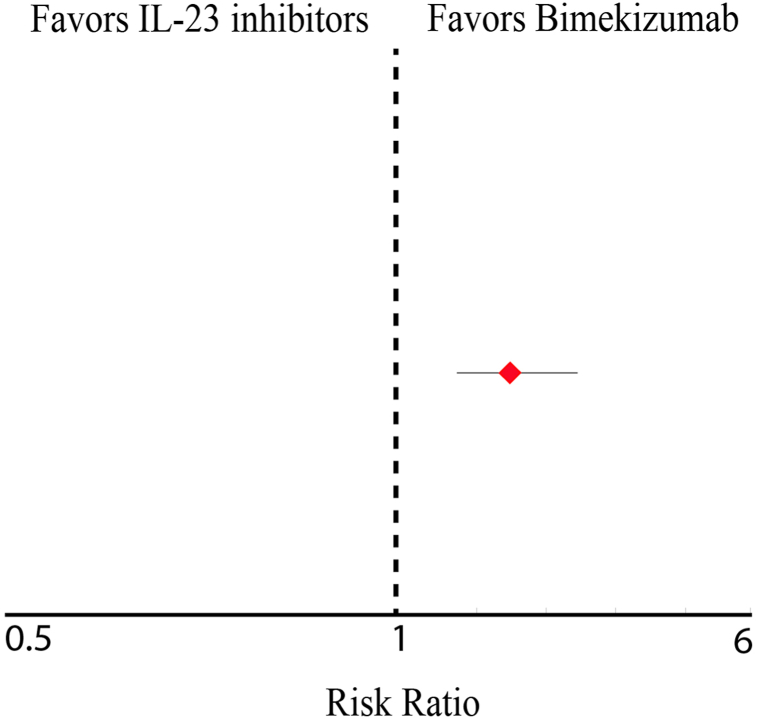
Forest plot comparing the risk of new-onset depression or suicidal ideation among adults with psoriasis treated with bimekizumab versus IL-23 inhibitors. Risk ratio = 2.43 (95% CI, 1.715-3.451; *P* < .001), indicating significantly higher psychiatric event rates among IL-23 users. *CI*, Confidence interval; *IL*, interleukin.

In a sensitivity analysis restricting both cohorts to a uniform 1-month to 6-month postindex window, bimekizumab was not associated with an increased risk of depression or SI compared with IL-23 inhibitors. The 6-month cumulative incidence of depression or SI was 4.0% among IL-23 inhibitor users and 2.7% among bimekizumab users (risk difference: 1.3%, 95% confidence interval: 0.004-0.031; *P* = .125).

Our findings suggest that bimekizumab is not associated with an increased risk of depression or SI relative to IL-23 inhibitors in patients with psoriasis in real-world clinical settings. These data align with pooled clinical-trial safety results for both IL-17 and IL-23 pathways, where psychiatric events occur rarely and at rates consistent with background psoriasis populations.^3^ Although causality cannot be inferred from observational data, the large multicenter dataset and robust matching strengthen confidence in these results. Routine mental-health screening remains essential in patients receiving biologic therapy; however, these findings provide reassurance that bimekizumab does not confer additional psychiatric risk relative to other advanced biologics.

## Conflicts of interest

Dr Leonardo Tjahjono has served as a consultant and/or speaker for Arcutis, Bristol Myers Squibb, Eli Lilly, Incyte, LEO Pharma, and Galderma. Dr Adam Friedman is in the consulting/ad board of La Roche Posay, Galderma, Kenvue, Microcures, LEO Pharma, Pfizer, Hoth Therapeutics, Zylo Therapeutics, Mino Labs, J&J, Arcutis, Lilly, UCB, Novartis, UCB, Regeneron/Sanofi, and Takeda; is a speaker for Regeneron/Sanofi, J&J, Incyte, UCB, Galderma, Arcutis, Lilly, Pfizer, and Novartis; Grants from Pfizer, Lilly, Galderma, Incyte, J&J, Abbvie, Loreal, and Regeneron/Sanofi. Dr Joaquin Calderon and Miss Christina Tolete have no conflicts of interest to declare.
